# Comparative proteomics provides insights into diapause program of *Bactrocera minax* (Diptera: Tephritidae)

**DOI:** 10.1371/journal.pone.0244493

**Published:** 2020-12-31

**Authors:** Jia Wang, Li-Lin Ran, Ying Li, Ying-Hong Liu

**Affiliations:** College of Plant Protection, Institute of Entomology, Southwest University, Chongqing, China; USDA Agricultural Research Service, UNITED STATES

## Abstract

The Chinese citrus fly, *Bactrocera minax*, is a notorious univoltine pest that causes damage to citrus. *B*. *minax* enters obligatory pupal diapause in each generation to resist harsh environmental conditions in winter. Despite the enormous efforts that have been made in the past decade, the understanding of pupal diapause of *B*. *minax* is currently still fragmentary. In this study, the 20-hydroxyecdysone solution and ethanol solvent was injected into newly-formed pupae to obtain non-diapause- (ND) and diapause-destined (D) pupae, respectively, and a comparative proteomics analysis between ND and D pupae was performed 1 and 15 d after injection. A total of 3,255 proteins were identified, of which 190 and 463 were found to be differentially abundant proteins (DAPs) in ND1 *vs* D1 and ND15 *vs* D15 comparisons, respectively. The reliability and accuracy of LFQ method was validated by qRT-PCR. Functional analyses of DAPs, including Gene Ontology (GO) and Kyoto Encyclopedia of Genes and Genomes (KEGG) enrichment, and protein-protein interaction (PPI) network construction, were conducted. The results revealed that the diapause program of *B*. *minax* is closely associated with several physiological activities, such as phosphorylation, chitin biosynthesis, autophagy, signaling pathways, endocytosis, skeletal muscle formation, protein metabolism, and core metabolic pathways of carbohydrate, amino acid, and lipid conversion. The findings of this study provide insights into diapause program of *B*. *minax* and lay a basis for further investigation into its underlying molecular mechanisms.

## Introduction

The Chinese citrus fly, *Bactrocera minax* (Enderlein), has long been recognized as one of the most notorious pests of citrus in temperate Asia, especially in China [1, 2]. The oligophagous *B*. *minax* specifically damage cultivated and wild species of citrus by larvae feeding inside the fruits which occasionally cause tremendous yield losses [2, 3]. Therefore, concerns have been raised over *B*. *minax* in citrus-growing regions in China. Accordingly, a number of studies focused on the ecology, biology, physiology, and management of *B*. *minax* have previously been carried out, so as to strengthen the comprehensive understanding of this pest [4–11].

Like numerous univoltine insects that enter obligatory diapause at a specific stage in each generation without requirement of token stimuli for diapause induction and preparation [12, 13], *B*. *minax* goes into obligatory pupal diapause each year to overwinter and synchronize adult emergence with citrus fruit bearing [14]. Diapause is a genetically programmed developmental arrest, featuring intense physiological alterations such as depressed metabolism and enhanced stress tolerance, in response to acute environmental stresses [12, 15]. Therefore, revealing the molecular mechanisms of *B*. *minax* pupal diapause will be conducive to elucidating the inherent mechanisms underlying its pupal development and adaptation to an adverse environment, and exploiting the potential of artificial regulation of diapause in pest management [16]. To this end, efforts have been made in the past decade to deepen the understanding of pupal diapause of *B*. *minax*. For example, the course of diapause was determined by constructing a respiratory rate trajectory throughout the pupal stage [17]. In addition, 20-hydroxyecdysone (20E) application on newly-formed pupae was found to be able to avert pupal diapause and significantly accelerate adult emergence [9, 18]. Given the complexity of physiological variations associated with initiation, maintenance, and termination of diapause, as well as the extensive diversity of diapause strategies across Insecta [19, 20], the high-throughput and large-scale -omics technologies have been regarded as ideal approaches to decipher the mechanisms underlying diapause in many insects [13, 19–23]. Likewise, these technologies have also been adopted to explore the diapause-associated physiological alterations in *B*. *minax* [17, 24–27]. Nevertheless, the understanding of mechanisms underlying its pupal diapause is currently still fragmentary.

Proteomics refers to the systematic identification and quantification of complete complement of proteins of biological systems. As proteins are the primary functional molecules in various physiological processes, proteomics is a promising alternative strategy to better reflect the physiological status of biological systems under specific conditions. A variety of quantitative proteomics methods have been developed and categorized into 2-dimensional gel electrophoresis (2-DE)-based and shotgun-based approaches [28, 29]. Given the ability to measure thousands of proteins and post-translational modifications in parallel, shotgun-based proteomics approaches, including label-free, metabolic labeling, and isobaric chemical labeling mass spectrometry (MS) methods, have fundamentally changed the way in which biological systems are investigated, and thus become the *de facto* standard for quantitative measurements in proteomics [29]. Recently, an increasing number of proteomics studies have been performed to reveal the inherent mechanisms underlying insect diapause, and remarkably expanded our knowledge in this field [30–34].

In this study, a comparative proteomics was performed using MS-based label-free quantitative proteomics technology to mine differentially abundant proteins (DAPs) between non-diapause- (ND) and diapause-destined (D) pupae of *B*. *minax*. Of MS-based proteomics technologies, the economical and labor-saving label-free method provides the deepest proteome coverage, but is not as efficient as the others in terms of accuracy, precision, and reproducibility [28]. Therefore, the proteomics data was subsequently verified by quantitative real-time PCR (qRT-PCR). The objectives of this study were to provide new insights into pupal diapause of *B*. *minax* and lay a basis for further relevant investigations.

## Materials and methods

### Ethics statement

Sample collection for our scientific research was permitted by the owner of an orchard in Wulong County, Chongqing Municipality, China.

### Insect rearing and sample collection

Large amounts of *B*. *minax* infested oranges were taken back to the lab from an orchard in Wulong County (N 29° 34.373', E 107° 54.564'), Chongqing Municipality, China. All oranges were peeled to collect the third-instar larvae, which were then placed over sand in plastic dishes to pupate. All pupae were reared at 18 ± 2°C, 70 ± 10% relative humidity, and photoperiod of 14 L:10 D.

### Acquisition of ND- and D-destined pupae

**Fig 1:**
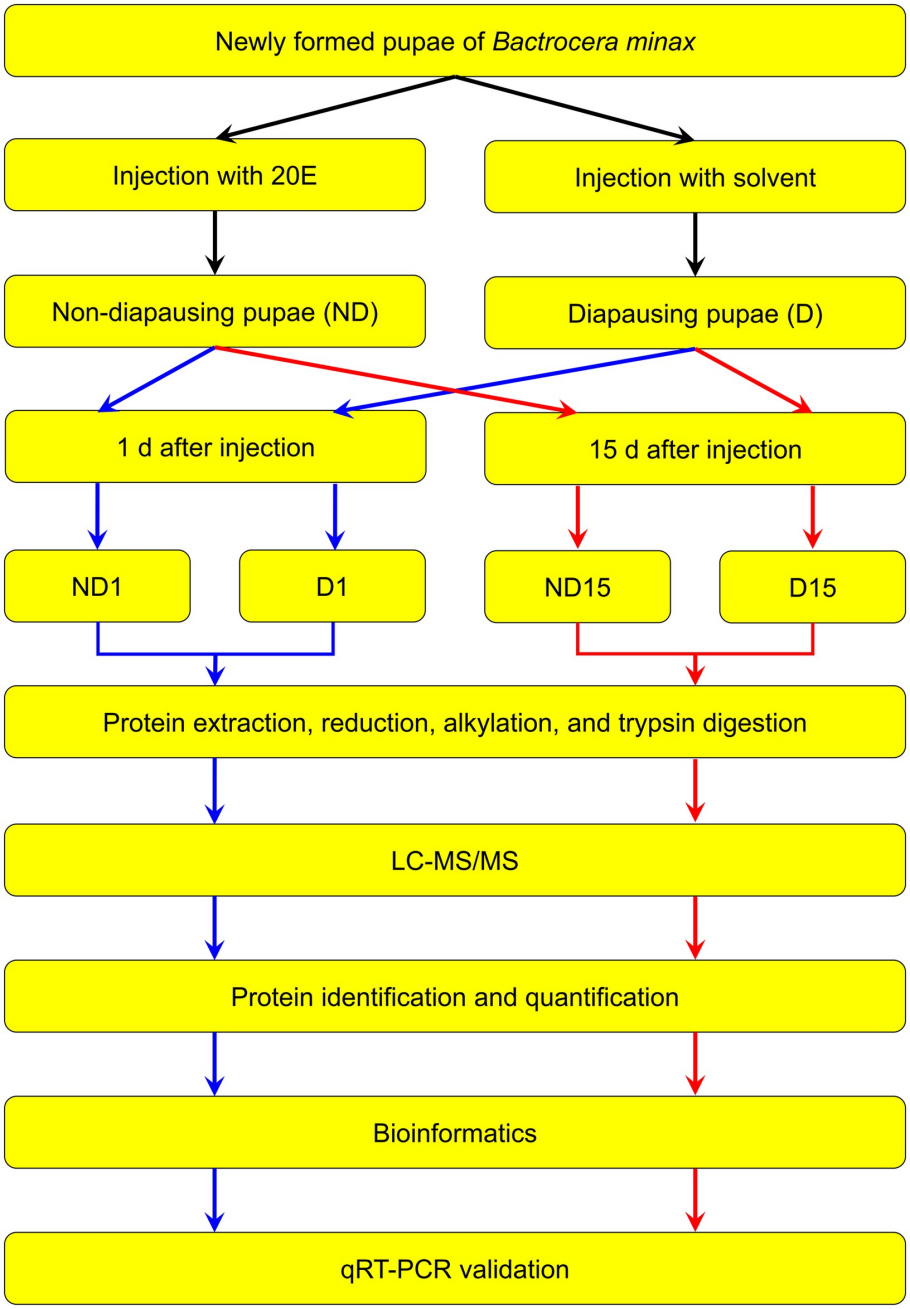
Diagram of workflow for comparative proteomics between non-diapause-(ND) and diapause-destined (D) pupae of *Bactrocera minax*. Blue and red arrows represent ND1 *vs* D1 and ND15 *vs* D15, respectively.

Two hundred newly-formed pupae (within 24 h after pupation) with the same size (~ 9 mm) were evenly divided into two groups for acquiring ND and D pupae, respectively. 20E (Sigma, St. Louis, MO, USA) was dissolved in 10% ethanol to concentration of 1 μg/μL. Each pupa in ND group was injected with 1 μL 20E solution to avert the diapause and acquire ND pupa, while each individual in the D group was injected with 1 μL 10% ethanol solvent to obtain D pupa [9, 26]. Then, all pupae were reared in plastic dishes under the same conditions described above. One and 15 d after injection, ND and D pupae were collected, referred to as ND1, D1, ND15, and D15 group (20 pupae for each group), respectively, and constantly stored in liquid nitrogen until subsequent comparative proteomics and qRT-PCR analysis between ND and D pupae (Fig 1).

### Protein extraction and digestion

Two individuals were randomly selected from 20 pupae in each group for protein extraction. The selected pupae were ground together in liquid nitrogen and homogenized in SDT buffer (4% SDS, 100 mM Tris-HCl, 1 mM DTT, pH 7.6). The homogenate was sonicated and boiled for 15 min. After centrifugation at 14000g for 40 min, the supernatant was filtered with 0.22 μm filter. The protein content of the filtrate was quantified using BCA Protein Assay Kit (Bio-Rad). Samples for MS measurement were prepared according to the filter-aided sample preparation (FASP) method [35]. Briefly, 200 μg of protein extraction was incorporated into 30 μL SDT buffer (4% SDS, 150 mM Tris-HCl, 100 mM DTT, pH 8.0) and cleaned by repeated ultrafiltration. Then, the cleaned protein filter was mixed with 100 μL 100 mM iodoacetamide solution dissolved in UA buffer (8 M Urea, 150 mM Tris-HCl, pH 8.0) and incubated in darkness for 30 min to block reduced cysteine residues. After washing with 100 μL UA buffer three times and 100 μL 25mM NH_4_HCO_3_ buffer twice, the protein suspension was digested by 4 μg trypsin in 40 μL 25mM NH_4_HCO_3_ buffer. The digested sample was desalted on C18 Cartridges (Empore^™^ SPE Cartridges C18 (standard density), bed I.D. 7 mm, volume 3 mL, Sigma) and concentrated by vacuum centrifugation, followed by reconstitution in 40 μL of 0.1% (v/v) formic acid for liquid chromatography-tandem mass spectrometry (LC-MS/MS) analysis. Three biological replicates were set for each group, and a total of 12 replicates for four groups were prepared for LC-MS/MS analysis.

### LC-MS/MS analysis

Each sample was subjected to high performance liquid chromatography (HPLC) separation using Easy nLC (Thermo Fisher Scientific, Waltham, MA, USA). The peptide mixture was loaded onto a C18 trap column (Thermo Scientific™ Acclaim™ PepMap™ 100, 100 μm × 2 cm, with nanoViper™ fingertight fitting) connected to a C18-reversed phase analytical column (Thermo Scientific Easy Column, 10 cm long, 75 μm inner diameter, 3μm resin) in buffer A (0.1% formic acid), and separated with a linear gradient of buffer B (84% acetonitrile and 0.1% Formic acid) at a flow rate of 300 nL/min for 2 h (0–55% buffer B for 110 min, 55–100% buffer B for 5 min, hold in 100% buffer B for 5 min). MS analysis was then performed on a Q Exactive mass spectrometer (Thermo Scientific) that was coupled to Easy nLC. The mass spectrometer was operated in positive ion mode. MS data was acquired using a data-dependent top 10 method that dynamically chose the most abundant precursor ions from the survey scan (300–1800 m/z) for high-energy collisional dissociation (HCD) fragmentation. Automatic gain control (AGC) target was set to 3e6, and maximum inject time to 10 ms. Dynamic exclusion duration was 40.0 s. Precursor ions that were singly-charged and unassigned were excluded. The full scan MS spectra and HCD spectra were acquired at a resolution of 70,000 and 17,500, respectively, at 200 m/z, and isolation width was 2 m/z. Normalized collision energy was 30 eV and the underfill ratio was defined as 0.1%. The instrument was run with peptide recognition mode enabled.

### Protein identification and quantification

The MS data were analyzed using MaxQuant software version 1. 5. 3. 17 (Max Planck Institute of Biochemistry in Martinsried, Germany) to identify the peptide sequences [36]. Two missed cleavages were allowed. Mass accuracy for the first and main search was 20 ppm and 6 ppm, respectively. Mass tolerance was 20 ppm for fragment ions. Fixed modifications were carbamidomethylation of cysteines, and variable modification was oxidation of methionine and N-terminal acetylation. Each HCD spectra was scored by Andromeda to reflect the quality of MS data [37]. A Pacbio sequencing full-length transcriptome (NCBI accession number: SRR12993052) and an assembled Illumina sequencing transcriptome (NCBI accession number: SRR1272962) of *B*. *minax* were merged and clustered. Then, unigenes were aligned to NCBI nr and Swiss-Prot database to deduce the amino acid sequences, and those failed to be matched against both database were analyzed by Transdecoder software to predict open reading frames (ORF). All these deduced amino acid sequences were lumped together as a protein database, against which the peptides identified by MS were searched. The cutoff of the false discovery rate (FDR) for peptide and protein was adjusted to 0.01. The mass spectrometry proteomics data have been upload to ProteomeXchange Consortium via the PRIDE [38] partner repository with the data set identifier PXD022576.

All identified proteins were quantified from normalized LFQ intensity. For each group, proteins with missing LFQ intensity in two replicates were removed in quantitative analysis. The relative levels of proteins between ND and D groups were expressed as LFQ intensity ratio (i.e. fold change), and the significance of difference was determined by Student’s t test using R. The criteria for identification of DAPs were defined as fold change > 2 and *p* < 0.05. In addition, proteins that were detected in at least two replicates of one group, but not in all three replicates of the other group, were deemed as DAPs as well.

### Bioinformatics analysis of DAPs

Clustering analysis of DAPs, excluding those only detected in either ND or D group, was performed using Cluster 3.0 (http://bonsai.hgc.jp/~mdehoon/software/cluster/software.htm) and visualized by Java Tree view [39]. Protein sequences of all DAPs were aligned against NCBI nr database and Swiss-Prot database using blastp in BLAST+ package (ncbi-blast-2.2.28+-win32.exe) to find homologue sequences. Gene Ontology (GO) mapping and annotation was performed using Blast2GO [40]. Then, all DAPs were blasted against the online Kyoto Encyclopedia of Genes and Genomes (KEGG) database (http://geneontology.org/) to retrieve their KEGG orthology identities and assignment of KEGG pathways. GO and KEGG Enrichment analyses were carried out using Fisher’ exact test in R, taking the whole quantified protein annotations as background dataset. Benjamini-Hochberg correction for multiple testing was further adopted to adjust derived *p*-values. Only functional categories and pathways with *p* < 0.05 were considered to be significantly changed. The PPI information of DAPs was retrieved from the IntAct molecular interaction database (http://www.ebi.ac.uk/intact/) by gene symbols or STRING (http://string-db.org/). The PPI networks were visualized using Cytoscape5 version 3.2.1 (http://www.cytoscape.org/).

### qRT-PCR validation

qRT-PCR was performed to validate the accuracy of the LFQ analysis. Total RNA was extracted from a randomly selected pupa in each group using TRIZOL Reagent (Life technologies, Carlsbad, CA, US). The first-strand cDNA was synthesized using PrimeScript^™^ RT Master Mix (Perfect Real Time) Kit (Takara, Shiga, Japan). Sixty-four pairs of specific primers were designed to amplify the randomly selected genes encoding DAPs determined by LFQ data (S1 Table), and *UBQ* was used as a reference gene [41]. qRT-PCR was performed with a CFX96^™^ Real-Time PCR Detection System thermal cycler (Bio-Rad, Hercules, CA, USA) in a reaction volume of 10 μL, containing 5 μL SYBR^®^ Premix Ex Taq II (Takara), 0.5 μL forward and reverse primers (10 μM), 1μL cDNA, and 3 μL ddH_2_O. The reaction conditions were as follows: 95°C for 30s; followed by 40 cycles of 95°C for 5s and 60°C for 30s. A relative standard curve was constructed based on a series of 10-fold diluted cDNA templates to estimate the amplification efficiency (E = 10^−1/slope^) of each pair of primers (S1 Table). Pearson’s r correlation coefficient was calculated to evaluate the correlation between the qRT-PCR and LFQ data. Three biological and three technical replicates were set for each gene.

## Results

### Quantitative overview of identified proteins

**Fig 2:**
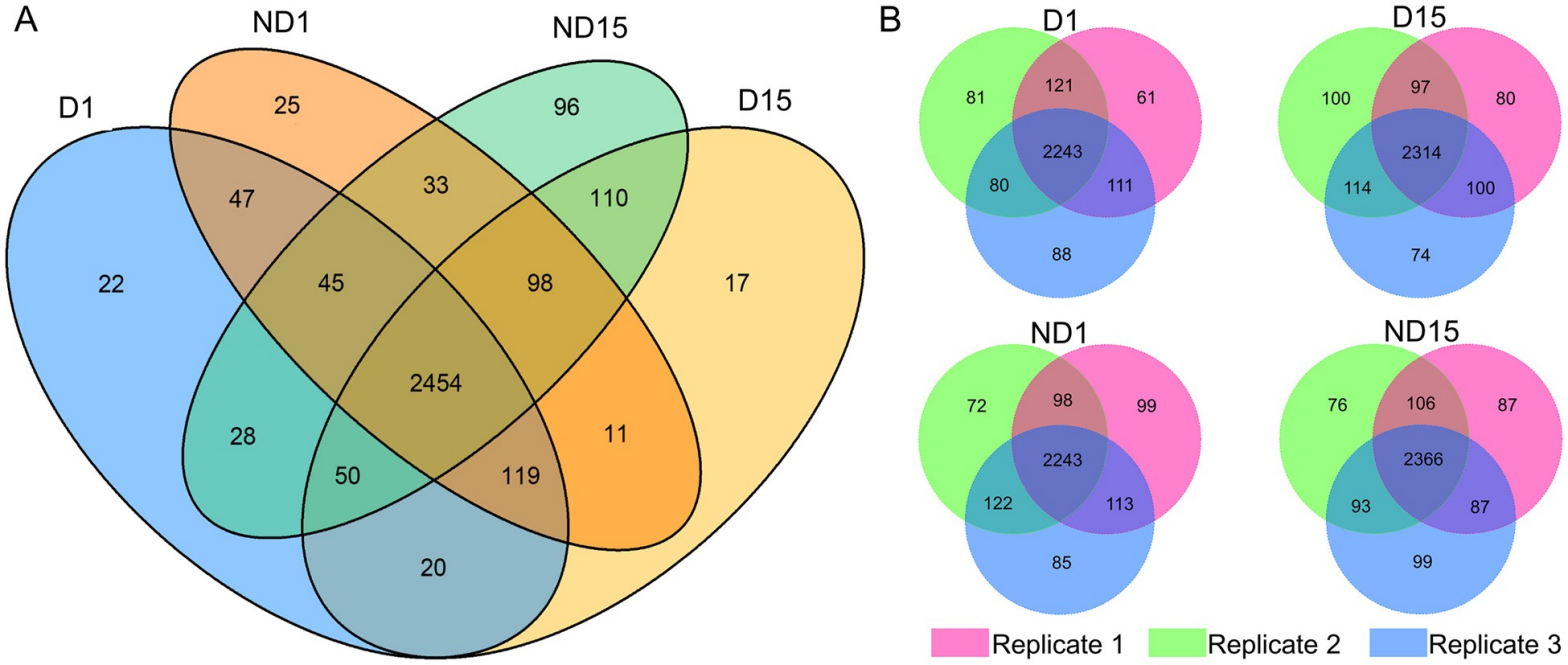
Venn diagram of proteins identified from non-diapause-(ND) and diapause-destined (D) pupae of *Bactrocera minax*. (A) Number of proteins identified from D1, D15, ND1, and ND15. The proteins identified from at least one of three replicates were shown in the diagram. (B) Number of proteins identified from three replicates in D1, D15, ND1, and ND15, respectively.

A total number of 12,237 peptides were identified from 12 samples (S2 Table), mapping onto 3,255 proteins (S3 Table) with molecular weight ranging from 2.727 to 436.45 kDa. Specifically, 2,785, 2,879, 2,832, and 2,914 proteins were derived from D1, D15, ND1, and ND15, respectively. Large overlaps of derived proteins could be observed in intra- and inter-group comparisons (Fig 2). The mass error distributed within 10 ppm (S1 Fig), indicating the accuracy of peptide identification. The median Andromeda score is 108.24 points, and 91.41% of peptides scored over 60 points (S2 Fig), reflecting the high quality of MS data.

**Fig 3:**
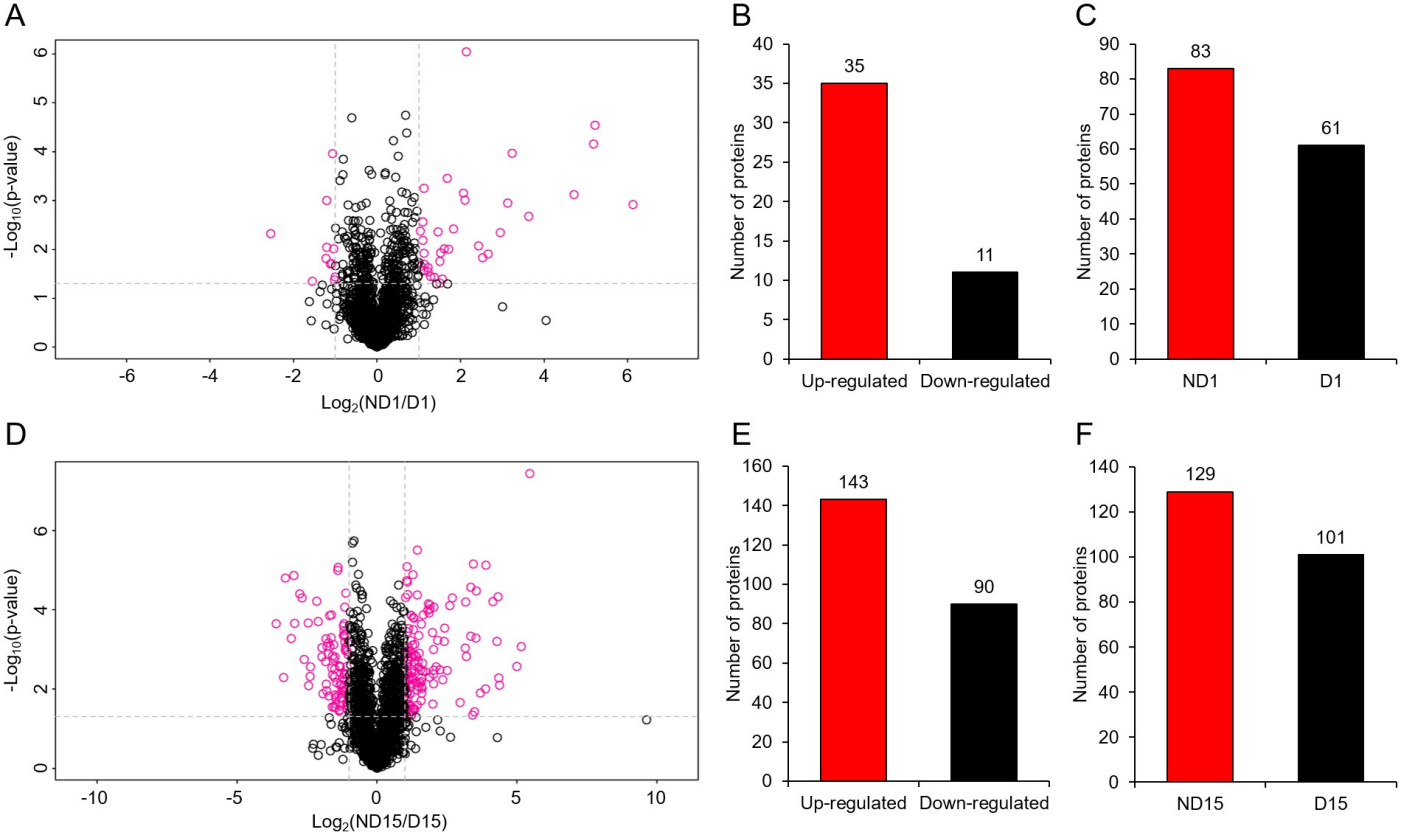
Comparisons of protein expression patterns between non-diapause-(ND) and diapause-destined (D) pupae of *Bactrocera minax*. (A-C) Differentially abundant proteins (DAPs) in ND1 *vs* D1 comparison. (D-F) DAPs in ND15 *vs* D15 comparison. (A and D) Volcano plot of log_2_ fold-change vs -log_10_ *p* value in each comparison. Fold change > 2 and *p* < 0.05 were set as the threshold for significant differential abundance. (B and E) Number of proteins that were detected in both ND and D groups with significantly different abundance. (C and F) Number of proteins that were.

**Fig 4:**
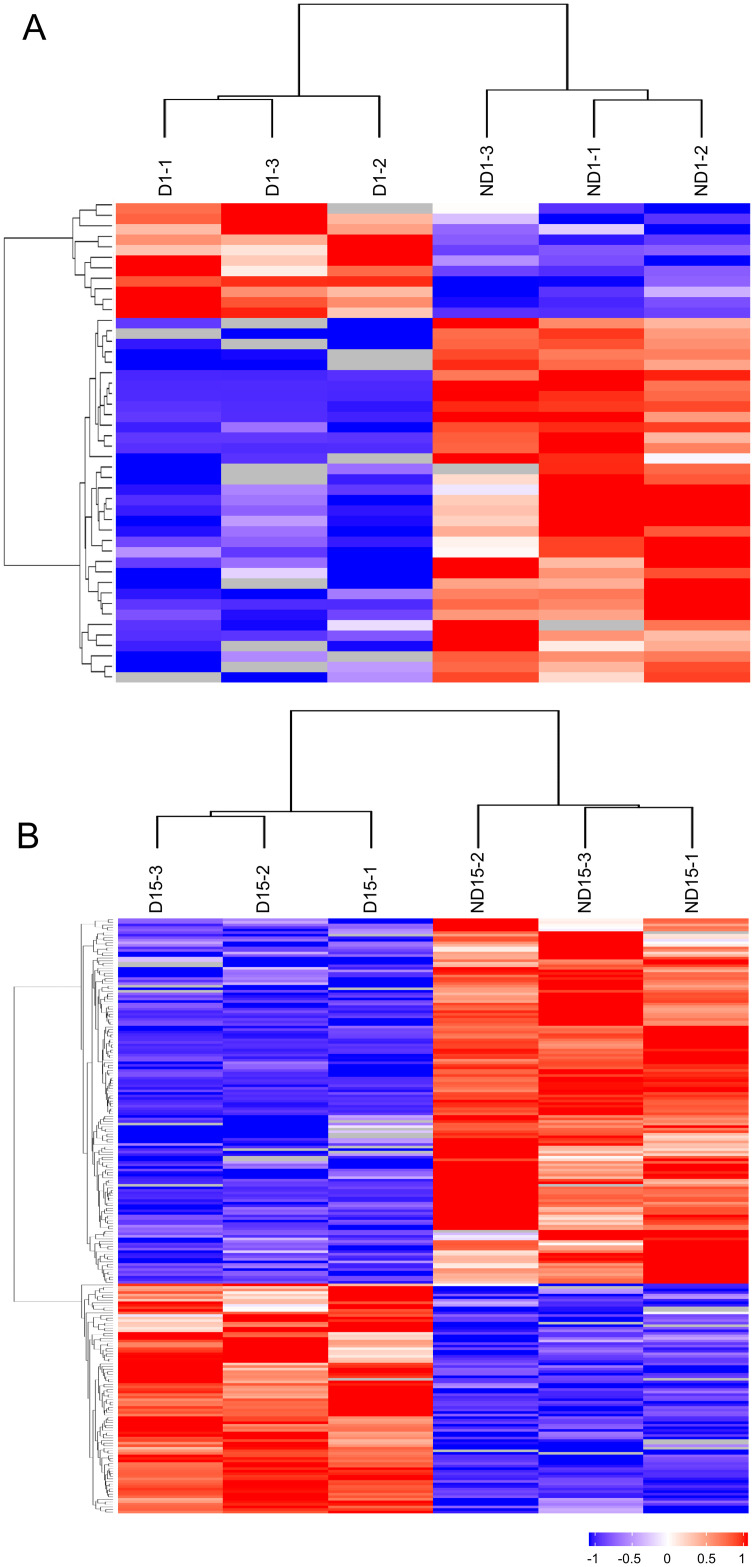
Clustering analysis of differentially abundant proteins (DAPs) between non-diapause-(ND) and diapause-destined (D) pupae of *Bactrocera minax*. (A) DAPs in ND1 *vs* D1 comparison. (B) DAPs in ND15 *vs* D15 comparison. Proteins that were detected in both ND and D groups with significantly different abundance were used, and those that were detected in at least two replicates of one group, but not in all three replicates of the other group, were excluded.

In ND1 *vs* D1 comparison, 190 DAPs were identified (S4 Table), including 46 proteins that were detected in both groups with significantly different abundance (35 up-regulated and 11 down-regulated in ND1) and 144 proteins that were only detected in either of two groups (83 in ND1 and 61 in D1) (Fig 3A–3C). In ND15 *vs* D15 comparison, 463 DAPs were identified (S4 Table), including 233 proteins that were detected in both groups with significantly different abundance (143 up-regulated and 90 down-regulated in ND15) and 230 proteins that were only detected in either of two groups (129 in ND15 and 101 in D15) (Fig 3D–3F). For each comparison, the relative abundance of DAPs that were detected in both groups were visualized by a heatmap (Fig 4). Additionally, the clustering analysis of DAPs showed that all replicates of the same group clustered together, indicating high similarity of protein profiles within groups (Fig 4).

### GO enrichment analysis of DAPs

**Fig 5:**
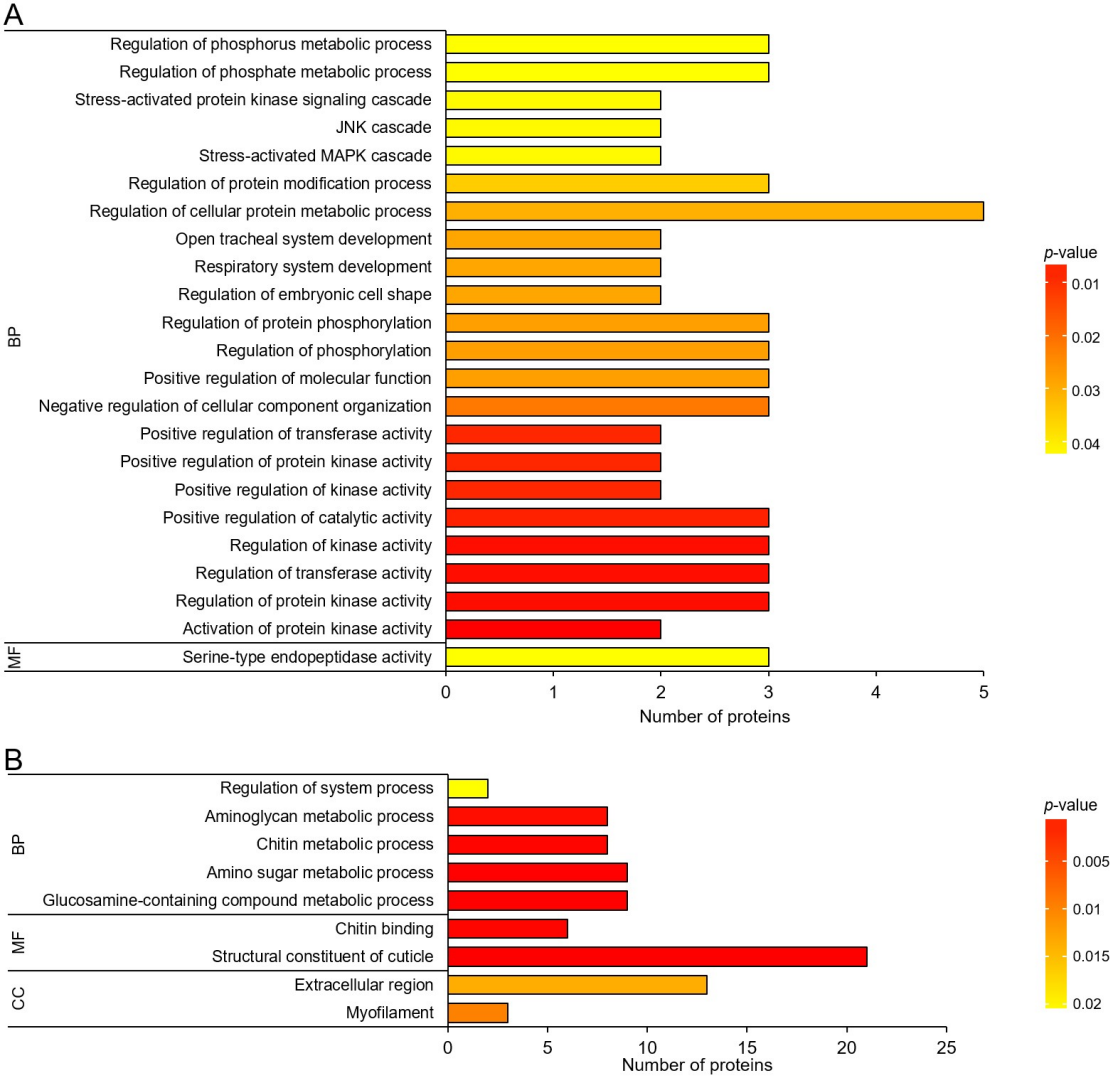
Gene ontology (GO) enrichment analysis of differentially abundant proteins (DAPs) between non-diapause-(ND) and diapause-destined (D) pupae of *Bactrocera minax*. (A) GO enrichment in ND1 *vs* D1 comparison. (B) GO enrichment in ND15 *vs* D15 comparison. GO terms fall into three main categories, cellular component (CC), molecular function (MF), and biological process (BP). The color gradient corresponds to the magnitude of *p* value.

Of 190 DAPs identified in ND1 *vs* D1 comparison, 74 proteins were assigned to GO terms composed of three main categories, cellular component (CC), molecular function (MF), and biological process (BP), and 23 GO functions were significantly differentiated. These GO functions were mainly involved in phosphorylation process (Table 1), signaling pathway, and development (Fig 5A and S5 Table). In addition, 180 out of 463 DAPs in ND15 *vs* D15 comparison were subcategorized into GO classes, and only 9 GO functions were significantly differentiated, which were related to substance metabolism, cuticle structure, and muscle structure (Fig 5B and S5 Table). DAPs involved with chitin metabolism were listed in Table 2.

**Table 1:** DAPs involved with phosphorylation in ND1 *vs* D1 comparison based on GO analysis.

Protein ID	Annotation	Regulation
c471656_g1	Serine/threonine-protein kinase mig-15	Down
c462764_g1	Ras-related protein Rac1	Up
c314836_g1	Casein kinase II beta subunit	Down

**Table 2:** DAPs involved with chitin metabolism in ND15 *vs* D15 comparison based on GO analysis.

Protein ID	Annotation	Regulation
c479709_g1	Chitinase-like protein Idgf3	Down
c112703_g1	Uncharacterized protein	Up
c247451_g1	Protein obstructor-E	Up
c448878_g1	Protein obstructor-E	Down
c465637_g1	Uncharacterized protein	Up
c467450_g1	Endochitinase	Up
c491125_g1	Protein obstructor-E	Up
c56657_g1	Hemocytin	Down

### KEGG pathway enrichment analysis of DAPs

**Fig 6:**
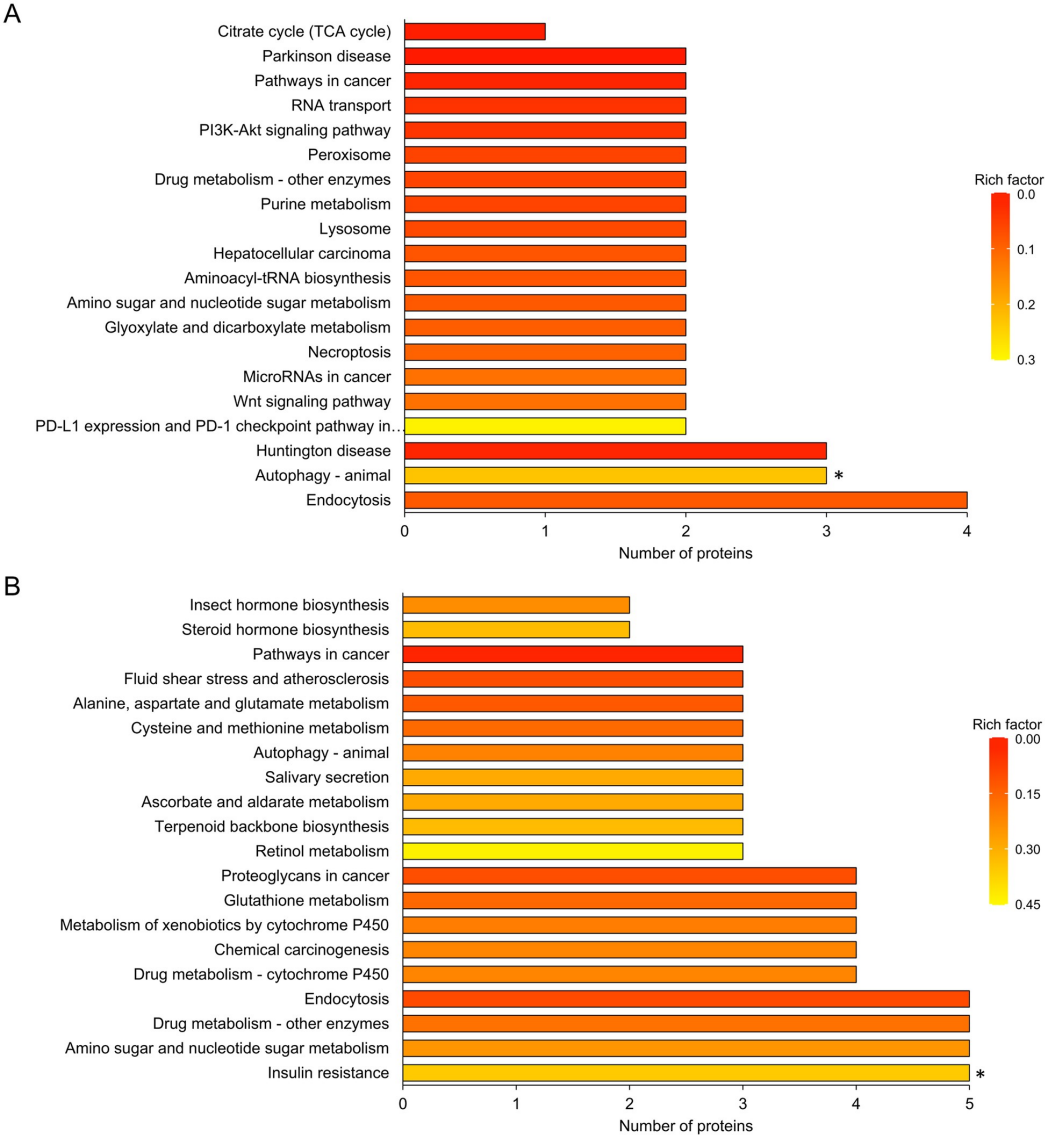
Kyoto Encyclopedia of Genes and Genomes (KEGG) enrichment analysis of differentially abundant proteins (DAPs) between non-diapause-(ND) and diapause-destined (D) pupae of *Bactrocera minax*. (A) KEGG enrichment in ND1 *vs* D1 comparison. (B) KEGG enrichment in ND15 *vs* D15 comparison. The color gradient corresponds to the magnitude of rich factor, which reflects the ratio of number of DAPs and all proteins that were assigned to the same pathway. Top 20 KEGG pathways in each comparison are shown. Asterisks indicate the significance of difference (*p* < 0.05).

KEGG pathway assignment was also performed for all DAPs between ND and D groups. In ND1 *vs* D1 comparison, 37 DAPs were assigned with 97 KEGG pathways, while only ‘Autophagy-animal’ pathway was significantly changed (Fig 6A and S6 Table). In ND15 *vs* D15 comparison, 75 DAPs were assigned with 136 KEGG pathways, while only ‘Insulin resistance’ pathway was significantly changed (Fig 6B and S6 Table). In both comparisons, ‘Endocytosis’ (Table 3), ‘Autophagy—animal’ (Table 4), ‘Amino sugar and nucleotide sugar metabolism’, ‘Drug metabolism—other enzymes’ and ‘Pathways in cancer’ pathways were among the top KEGG enrichments. Moreover, DAPs involved with Wnt signaling pathway in ND1 *vs* D1 comparison were listed in Table 5.

**Table 3:** DAPs involved with endocytosis in ND *vs* D comparisons based on KEGG analysis.

Protein ID	Annotation	Regulation	
		ND1 *vs* D1	ND15 *vs* D15
c456181_g1	Tumor susceptibility gene 101 protein	Up	Up
c499601_g1	Spartin	Up	Up
c479825_g1	Clathrin	Up	N. s.^a^
c514756_g1	Vacuolar protein sorting-associated protein 4A	Down	N. s.
c347997_g1	Vacuolar protein sorting-associated protein 35	N. s.	Up
c535763_g1	Sorting nexin-6	N. s.	Up
c555561_g1	Myc box-dependent-interacting protein	N. s.	Down

^a^ N. s. indicates that the difference of protein levels in this comparison was not significant.

**Table 4:** DAPs involved with autophagy in ND *vs* D comparisons based on KEGG analysis.

Protein ID	Annotation	Regulation	
		ND1 *vs* D1	ND15 *vs* D15
c116594_g1	Ubiquitin-like-conjugating enzyme ATG3	Up	Down
c486329_g1	Intrastrand cross-link recognition protein	Up	N. s.^a^
c472096_g1	Phosphatidylinositol-3,4,5-trisphosphate 3-phosphatase and dual-specificity protein phosphatase PTEN	Up	N. s.
c498830_g1	Ras-like protein 2	N. s.	Down
c553434_g1	Cathepsin L	N. s.	Up

^a^ N. s. indicates that the difference of protein levels in this comparison was not significant.

**Table 5:** DAPs involved with Wnt signaling in ND1 *vs* D1 comparison based on KEGG analysis.

Protein ID	Annotation	Regulation
c314836_g1	Casein kinase II beta subunit	Down
c527529_g1	RuvB-like helicase 1	Down

### Construction of PPI network for DAPs

**Fig 7:**
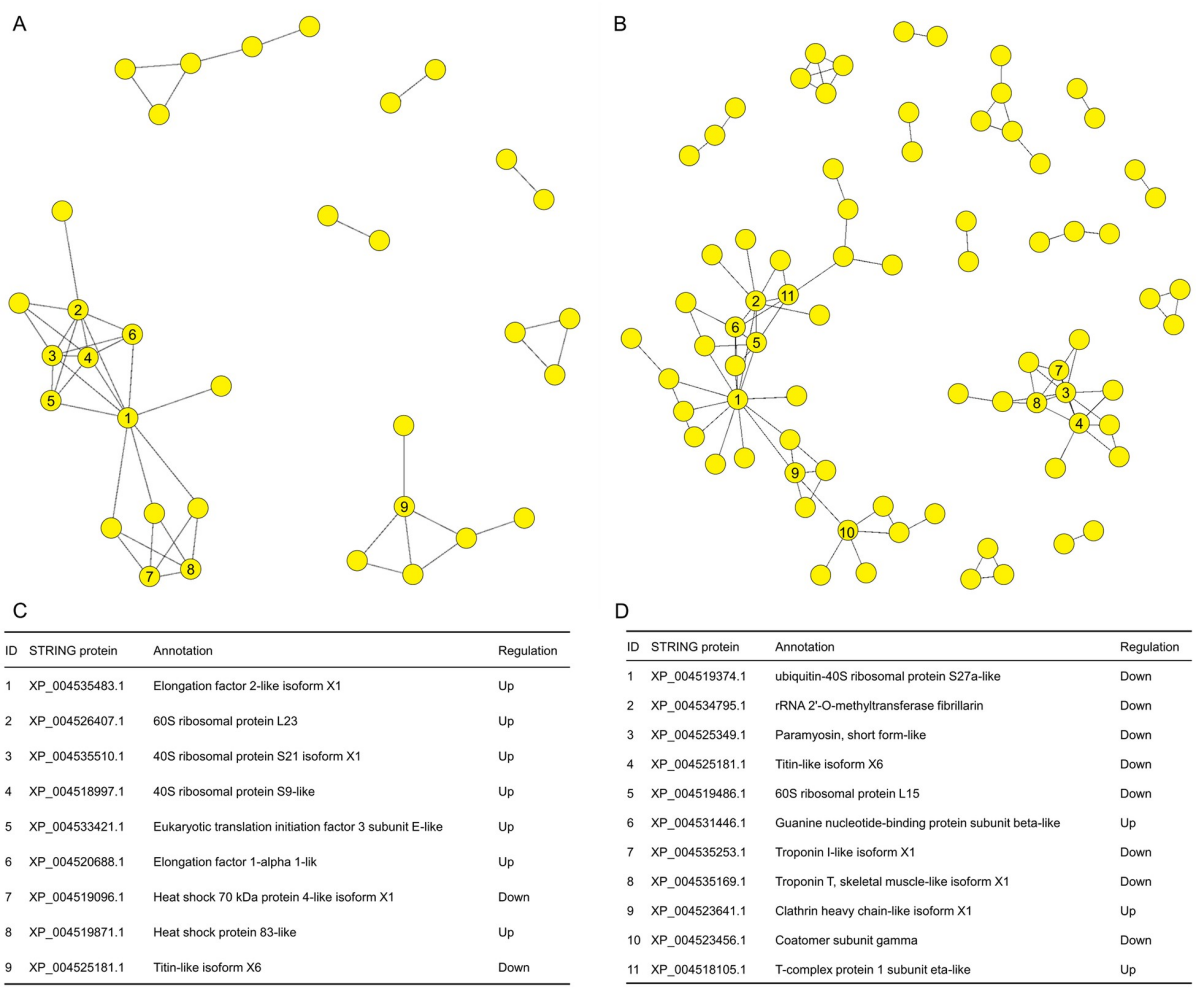
Protein-protein interaction (PPI) information of differentially abundant proteins (DAPs) between non-diapause-(ND) and diapause-destined (D) pupae of *Bactrocera minax*. (A and C) PPI information in ND1 *vs* D1 comparison. (B and D) PPI information in ND15 *vs* D15 comparison. (A and B) PPI networks. (C and D) Information of DAPs connected with at least four nodes in each PPI network.

For each comparison, a PPI network was constructed to illustrate the correlations of DAPs and crosslinks among biological pathways. In ND1 *vs* D1 comparison, 9 densely connected proteins (connected with at least four nodes) were found and fell into two clusters (Fig 7A and 7C). In ND15 *vs* D15 comparison, 11 densely connected proteins were identified and assigned to two clusters as well (Fig 7B and 7D). In both comparisons, two densely connected clusters are closely associated with function of protein metabolism and transport, and skeletal muscle formation, respectively.

### qRT-PCR validation

**Fig 8:**
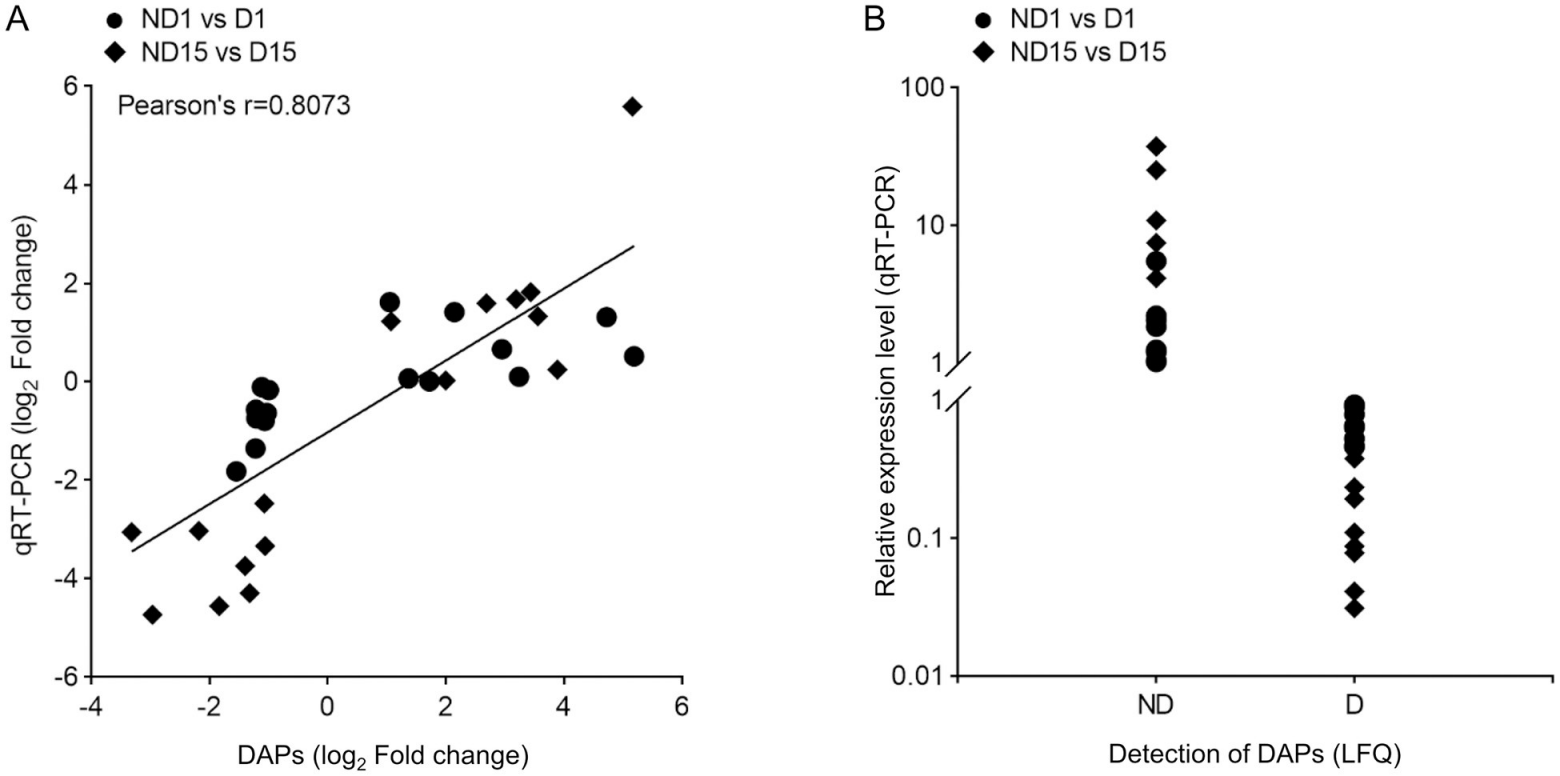
Validation of differentially abundant proteins (DAPs) between non-diapause-(ND) and diapause-destined (D) pupae of *Bactrocera minax* by qRT-PCR. (A) Pearson’s correlation analysis of qRT-PCR and mass spectrometry (MS)-based label-free quantification (LFQ) data for randomly selected DAPs that were detected in both ND and D groups by MS measurement; (B) Relative expression levels of genes encoding randomly selected DAPs that were only detected in either ND or D group by MS measurement.

Totally, 64 genes encoding DAPs determined by LFQ analysis were randomly selected to measure their relative abundance in two ND *vs* D comparisons using qRT-PCR. A strong correlation of qRT-PCR and LFQ data was shown (Fig 8), indicating the reliability of using MS-based LFQ method to investigate the protein expression profiles between ND and D pupae of *B*. *minax*.

## Discussion

To survive an adverse environment in summer/winter, insects have evolved a sophisticated diapause strategy, a programmed developmental arrest during specific life stages. Commonly, insects initiate and terminate diapause by exploiting environmental cues such as day length (facultative diapause), but in some cases, especially for univoltine insects, diapause occurs in each generation regardless of the prevailing environmental conditions (obligatory diapause) [12]. Diapause research was reckoned to be beneficiary in several areas, such as understanding the seasonal biology, developing effective pest management strategies, probing fundamental questions in development, and even providing insights into aging, obesity, and disease transmission of human [16]. Given the importance of diapause, substantial progress has been made in understanding details of diapause [12, 15, 16, 42]. Previously, the transcriptomics and metabolomics profiles along with pupal development of *B*. *minax* was investigated, and most of the variation was found to be related to metabolic pathways of carbohydrates, proteins, amino acids, and lipids conversion, as well as signaling pathways [17]. In this study, to enrich our knowledge of diapause of *B*. *minax*, the comparative proteomics between ND and D pupae was performed using MS-based LFQ method.

Of 3,255 proteins identified from 12 samples (Fig 2), hundreds of DAPs were discovered in two ND *vs* D comparisons (Fig 3), and were verified by qRT-PCR (Fig 8). The functional analysis of DAPs indicated that the diapause program of *B*. *minax* is closely associated with plenty of physiological activities, most of which are involved with core metabolic pathways of carbohydrates, proteins, amino acids, and lipids conversion (Figs 5 and 6). It is in line with the findings of our previous transcriptomics and metabolomics study [17], and the fact that metabolism is intensely depressed during insect diapause [12, 15]. In addition, physiological activities related to phosphorylation, chitin metabolism (Fig 5), autophagy, signaling pathways, endocytosis (Fig 6), and skeletal muscle formation (Fig 7), were also found to be changed during diapause of *B*. *minax*, demonstrating the significance of proteomics as a complementary approach to decipher the mechanisms underlying complex process of diapause.

Phosphorylation is a common chemical process in which a phosphate group released from ATP is added to a compound within the cell. Normally, the phosphorylation reaction is catalyzed by enzymes referred to as kinases that play crucial roles in various signaling pathways and regulatory mechanisms via modification of the protein structure and alteration of their activities [43, 44]. It has been demonstrated that the phosphorylation modifications participate in the regulation of insect diapause [45–49]. For example, in silkworm *Bombyx mori*, the temporal changes in glycogen synthase kinase-3β (GSK-3β) phosphorylation were closely related to changes in glycogen levels, showing an abrupt decrease at the onset of egg diapause and a great increase after chilling, while those of non-diapause-destined eggs remain relatively high [47]. In this study, several GO functions and KEGG pathways involved with phosphorylation modifications were enriched as well, implying that phosphorylation modifications also regulate the diapause of *B*. *minax* in certain ways, although the exact roles they play remain unknown.

Chitin, one of the most important biopolymers in nature, is produced in abundance by fungi, arthropods and nematodes. In insects, chitin is crucial for growth, development, and morphogenesis, owing to its function as scaffold material to support epidermis and trachea, as well as being a constituent part of the peritrophic matrices that line the inner surface of the gut to protect the intestinal epithelium from mechanical disruption, radical oxygen species and microorganisms invasion [50, 51]. Therefore, it was understandable that the chitin metabolic pathway was significantly changed in ND pupae as a large amount of chitin was required for development. In addition, it has been previously demonstrated that the chitin biosynthesis pathway in insects could be activated by 20E signaling via microRNAs modulation [52, 53], implying that the injected exogenous 20E *per se*, but not the averted diapause, regulated the chitin metabolism in ND pupae of *B*. *minax*.

Autophagy is a critical conserved process by which the cytoplasmic components, such as macromolecules and organelles, are degraded in lysosomes or vacuoles and recycled in response to a wide variety of physiological activities, including tissue remodeling, organelle quality control, and stress resistance [54–56]. To date, the direct evidence for linkage between autophagy and insect diapause is still scarce. However, autophagy has been demonstrated to play roles in the diapause process of other organisms, such as crustacean *Artemia parthenogenetica* [55] and harvestmen *Amilenus aurantiacus* [56]. Therefore, it is very interesting to consider that autophagy plays a role in insect diapause, which necessitates further verification.

It has been well-documented that the signaling pathways, for instance, insulin (IS), target of rapamycin (TOR), and Wnt signaling pathway, regulate the insect diapause [13, 19, 21, 57]. Active IS and TOR pathways promote cell proliferation and organismal growth [58]. Therefore, inactivation of IS and TOR pathways have often been observed in insect diapause, and linked to the suppression of growth/development and enhanced stress response [19, 22]. In this study, a few rearrangements in IS and TOR pathways between ND and D pupae of *B*. *minax* were observed, probably because many elements of the IS and TOR cascades are regulated via phosphorylation rather than by transcription [21], which is consistent with the significant changes in phosphorylation mentioned above. The highly conserved and complicated Wnt signaling is one of the most important developmental signaling pathways that controls cell fate decisions and tissue patterning. The Wnt signaling is composed of three related but distinct sub-pathways [59], and seems to be a regulator of insect diapause [13, 57]. In the present study, DAPs enriched in Wnt signaling pathway indicated that it is a candidate pathway for regulating diapause of *B*. *minax*.

Endocytosis is a process for cells to absorb nutrients and other substances which are necessary for cell health but too large to pass through the cell membrane. Many distinct endocytic pathways have been discovered in organisms and play key roles in a lot of physiological activities, for instance, nutrient uptake, cell signaling, cell shape changes, immunity, and pathogen internalization [60, 61]. So far, investigations on the correlation between endocytosis and insect diapause are still rare events, and only a few such case studies are available. For example, in diapausing *Drosophila melanogaster*, a pre-vitellogenic arrest of ovarian development is associated with the absence of clathrin, one of the key elements for endocytosis process [62]. It is not surprising that the endocytosis is involved with the insect diapause due to its multifunctional nature. Presumably, the activation of endocytosis in ND pupae was a concomitant of restored metabolism and augmented signaling.

During the process of pupa-adult metamorphosis, insects are undergoing histolysis and histogenesis, by which the organs and tissues are remodeling. Compared to D pupae of *B*. *minax*, ND pupae was maintaining development and preparing for metamorphosis [18]. Therefore, the loss of paramyosin, titin, and troponin in ND pupae is likely to be the consequence of muscle histolysis [63]. Additionally, the muscle wasting was regulated by several crucial genes, including TOR and AMPK [63], which is in agreement with the idea that TOR signaling pathway and phosphorylation are closely associated with diapause of *B*. *minax*.

## Conclusion

Despite the substantial progress that has been made recently, the understanding of diapause of *B*. *minax* was still fragmentary. In this study, the comparative proteomics between ND and D pupae was conducted, using MS-based LFQ method, as a complementary approach to decipher the mechanisms underlying diapause of *B*. *minax*. DAPs in two ND *vs* D comparisons were determined and enriched in several physiological activities, for instance, phosphorylation, chitin metabolism, autophagy, signaling pathways, endocytosis, skeletal muscle formation, protein metabolism, and core metabolic pathways of carbohydrate, amino acids, and lipids conversion. The findings provide insights into diapause program of *B*. *minax* and lay a basis for further investigation into its underlying molecular mechanisms.

## Supporting information

S1 Fig Mass error distribution of identified peptides from all samples of *Bactrocera minax* by MS detection. (TIF)

S2 Fig Andromeda score distribution of identified peptides from all samples of *Bactrocera minax* by MS detection. (TIF)

S1 Table Information of primers for qRT-PCR validation of genes encoding selected DAPs determined by LFQ analysis. (XLS)

S2 Table Peptides identified from all samples of *Bactrocera minax* by MS detection. (XLS)

S3 Table Proteins determined by peptides mapping onto the deduced amino acids sequences based on the full-length transcriptome of *Bactrocera minax*. (XLS)

S4 Table DAPs between ND and D pupae of *Bactrocera minax* determined by LFQ analysis. (XLS)

S5 Table Significantly differentiated Gene ontology (GO) functions between non-diapause-(ND) and diapause-destined (D) pupae of *Bactrocera minax*. (XLS)

S6 Table Differentiated Kyoto Encyclopedia of Genes and Genomes (KEGG) pathways between non-diapause-(ND) and diapause-destined (D) pupae of *Bactrocera minax*. (XLS)
