# Supplementary figures and images for: Comparative proteomics provides insights into diapause program of Bactrocera minax (Diptera: Tephritidae)
Source: PLoS One. 2020 Dec 31;15(12):e0244493. doi: 10.1371/journal.pone.0244493 (PMC7774860; doi:10.1371/journal.pone.0244493)

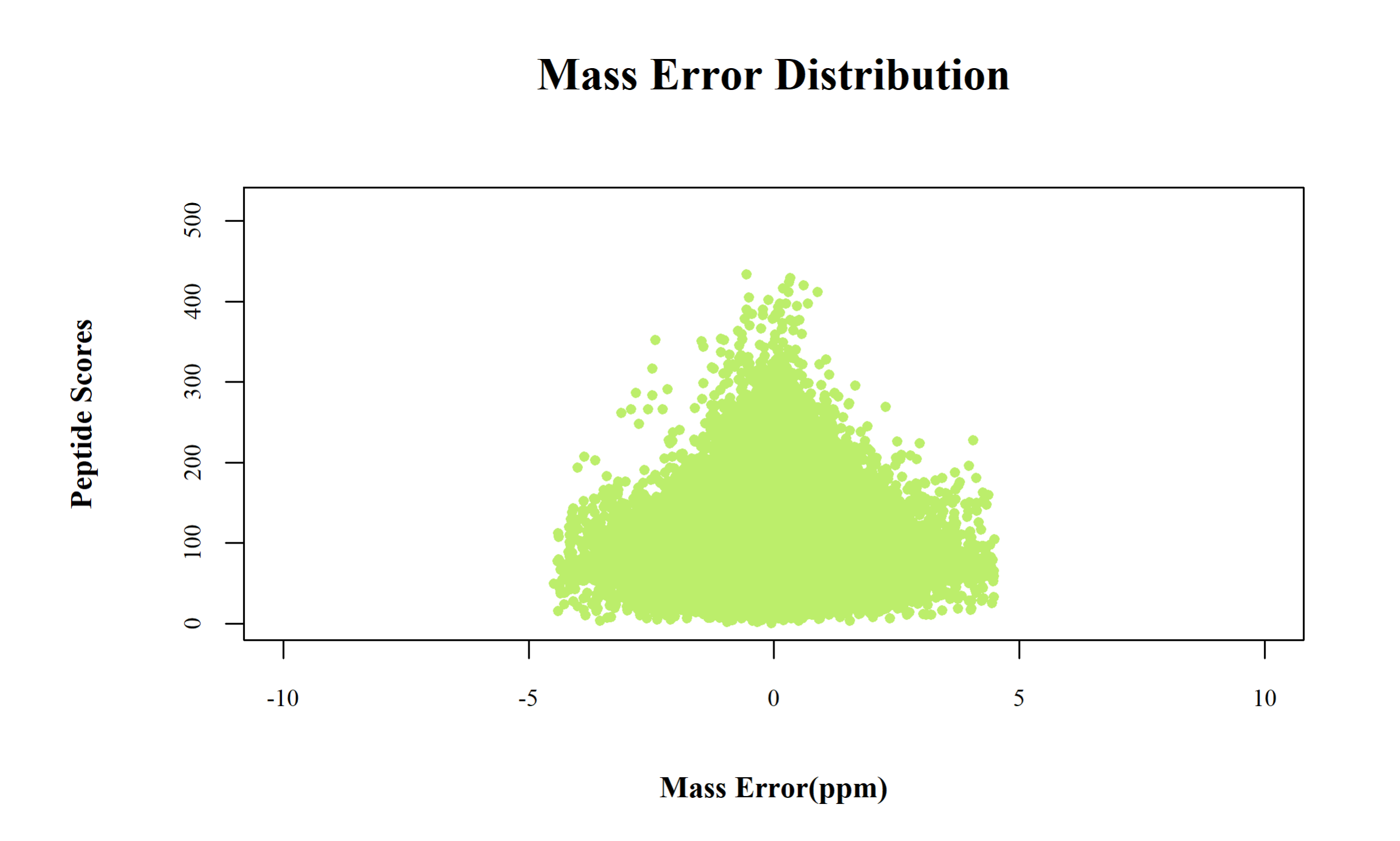

Supplement: S1 Fig — (TIF) [file pone.0244493.s001.tif]
